# A Questionnaire for Assessing User Satisfaction With Mobile Health Apps: Development Using Rasch Measurement Theory

**DOI:** 10.2196/15909

**Published:** 2020-05-26

**Authors:** Jeanette Melin, Stephanie Erika Bonn, Leslie Pendrill, Ylva Trolle Lagerros

**Affiliations:** 1 Research Institutes of Sweden AB Göteborg Sweden; 2 Clinical Epidemiology Division Department of Medicine Solna Karolinska Institutet Stockholm Sweden

**Keywords:** cell phone, healthy lifestyle, methods, mobile applications, psychometrics, smartphone, telemedicine, mobile phone

## Abstract

**Background:**

Mobile health (mHealth) apps offer great opportunities to deliver large-scale, cost-efficient digital solutions for implementing lifestyle changes. Furthermore, many mHealth apps act as medical devices. Yet, there is little research on how to assess user satisfaction with an mHealth solution.

**Objective:**

This study presents the development of the mHealth Satisfaction Questionnaire and evaluates its measurement properties.

**Methods:**

Respondents who took part in the Health Integrator Study and were randomized to use the Health Integrator smartphone app for lifestyle changes (n=112), with and without additional telephone coaching, rated their satisfaction with the app using the new 14-item mHealth Satisfaction Questionnaire. The ratings were given on a 5-point Likert scale and measurement properties were evaluated using Rasch measurement theory (RMT).

**Results:**

Optimal scoring was reached when response options 2, 3, and 4 were collapsed, giving three response categories. After omitting two items that did not fit into the scale, fit residuals were within, or close to, the recommended range of ±2.5. There was no differential item functioning between intervention group, age group, or sex. The Person Separation Index was 0.79, indicating that the scale’s ability to discriminate correctly between person leniency was acceptable for group comparisons but not for individual evaluations. The scale did not meet the criterion of unidimensionality; 16.1% (18/112) of the respondents were outside the desired range of −1.96 to 1.96. In addition, several items showed local dependency and three underlying dimensions emerged: negative experiences, positive experiences, and lifestyle consequences of using the mHealth solution.

**Conclusions:**

In times where mHealth apps and digital solutions are given more attention, the mHealth Satisfaction Questionnaire provides a new possibility to measure user satisfaction to ensure usability and improve development of new apps. Our study is one of only a few cases where RMT has been used to evaluate the usability of such an instrument. There is, though, a need for further development of the mHealth Satisfaction Questionnaire, including the addition of more items and consideration of further response options. The mHealth Satisfaction Questionnaire should also be evaluated in a larger sample and with other mHealth apps and in other contexts.

**Trial Registration:**

ClinicalTrials.gov NCT03579342; http://clinicaltrials.gov/ct2/show/NCT03579342.

## Introduction

### Background

Electronic health (eHealth), defined by the World Health Organization as the use of information and communication technologies for health, also encompasses mobile health (mHealth), defined as a medical or public health practice that is supported by mobile devices [1]. mHealth has had a rapid evolution and adoption and today, smartphone apps have the potential to make the treatment and prevention of diseases cost-efficient and widely accessible. There is a vast number of apps for tracking different types of health data such as physical activity, diet, sleep, stress, and more. Apps also commonly act as medical devices or accessories to medical devices to, for example, apps diagnose heart rhythm abnormalities or function together with a glucose meter used by an insulin-dependent patient with diabetes. The mHealth literature has grown rapidly over the last couple of years and has so far primarily focused on the effectiveness and efficiency of the usability of mHealth solutions.

While effectiveness refers to the completeness of specified goals, such as improved health status, which is often measured in terms of medical examinations and self-reported health, efficiency on the other hand, relates to the resources used for accomplishment, such as cost for personnel or digital solutions, rather than to its implications. However, from a full usability perspective, effectiveness and efficiency are important, but given the often high attrition rates in mHealth studies [2], user satisfaction may be key for retention.

To enable more comprehensive evaluations of mHealth solutions, there is a need to develop a questionnaire to assess user satisfaction. To the best of our knowledge, there is no generic commonly available tool, for example, a questionnaire, for capturing user satisfaction with mHealth solutions using an app. However, King et al [3] constructed a user satisfaction survey following their 8-week feasibility testing of different physical activity apps. It consisted of 22 items asking the respondents to rate usability on a 6-point Likert-type scale. The survey was subsequently adapted by Mummah et al [4] into a 21-item questionnaire in which participants were asked to rate their level of agreement or disagreement with different statements on a 5-point Likert-type scale. This questionnaire was used to measure user satisfaction of Vegathon, an app aiming to increase vegetable intake among adults with obesity. Statements in the questionnaires by King et al [3] and Mummah et al [4], as well as in the adapted version used here, include areas of usability such as time consumption, motivation, understanding, and willingness to recommend it.

### Objectives

In this work, we have developed a generic 14-item version called the mHealth Satisfaction Questionnaire. We used the new questionnaire in a large randomized controlled mHealth trial, the Health Integrator Study [5]. Here, we report how we used psychometric Rasch measurement theory (RMT) to assess the ability of our new mHealth Satisfaction Questionnaire to measure user satisfaction with an mHealth solution. On the basis of the results, we also suggest improvements for a future mHealth Satisfaction Questionnaire.

## Methods

### Data Collection

The study population comprised the respondents taking part in the Health Integrator Study’s intervention groups (n=138). Briefly, the 3-month interventions included a personalized mHealth intervention based on the participant’s personal health profile and were tailored to the need of each specific participant, with or without telephone sessions with a health coach. The intervention has been described in detail elsewhere [5]. The study was approved by the Regional Ethical Review Board in Stockholm, Sweden (2018/411-31 and 2018/1038-32).

**Figure 1:**
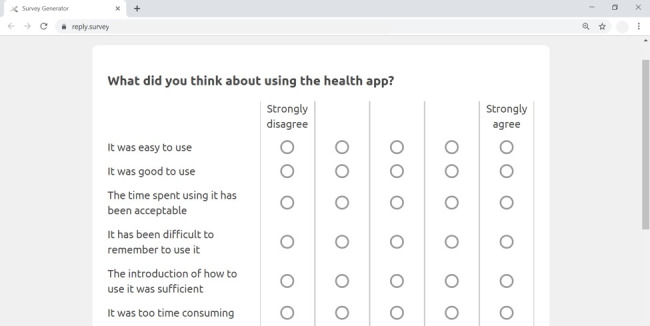
Screenshot of the Web-based mHealth Satisfaction Questionnaire.

At the 3-month follow-up of the active mHealth intervention, the participants were administered the Web-based mHealth Satisfaction Questionnaire, evaluating how the Health Integrator app was perceived—both in terms of usage and improved health. An example of the Web-based version of the questionnaire is shown in Figure 1. A reminder was sent after 2 weeks to all nonresponders.

In total, 112 respondents completed the new mHealth Satisfaction Questionnaire after the intervention, corresponding to a response rate of 81%. Of these 112 respondents, 60 (53.5%) received telephone support with a health coach during the active intervention, while 52 (46.4%) did not receive this support. The study population comprised a slightly greater number of men, 56.3% (63/112), than women, 43.8% (49/112). The mean age of participants was 47.8 years (median 48.5 years, range 26-73 years).

### Measurement

The mHealth Satisfaction Questionnaire is an adaption of the user satisfaction survey used in the Vegathon study by Mummah et al [4], which in turn is an adaption of the user satisfaction survey by King et al [3]. In adjusting the questionnaire to a short and more generic 14-item version, we omitted some of the more vegetable-specific questions used in the Vegathon study such as *Vegathon has given me the confidence that I could become a better vegetable eater*. Although Mummah et al [4] specifically targeted the vegetable intake app in each statement, our questionnaire is divided into two sections with the overarching questions: *What did you think about using the health app?* and *How did you experience the health app?* The sections of our questionnaire include general statements about, for example, the usability of and willingness to recommend the app.

The mHealth Satisfaction Questionnaire consists of 14 items where the respondent is asked to rate to what extent he or she agrees on each item on a 5-point Likert-scale (see Multimedia Appendix 1). Higher rating corresponds to higher agreement (ie, 1=strongly disagree, 5=strongly agree); 10 items are positively stated, while four items are negatively stated. The negatively stated items were reversed in the analyses, and consequently, higher values correspond to higher leniency.

Measurements made with any questionnaire need to produce results which are invariant and reliable in much the same way as is required of physical measurements, for instance of mass or length [6]. To ensure equitability and fairness, irrespective of to whom the questionnaire is administered, estimates of person and item characteristics (leniency and quality in this case) deduced from questionnaire responses need to be comparable, as far as possible, with corresponding estimates made on other occasions by other independent measurements. Second, any measurement result will have limited quality as there is neither time nor resources to perform perfect and complete measurements. The risks of incorrect decisions (about for instance care) associated with this uncertainty can be assessed if measurement reliability is openly declared in terms of how much uncertainty there is in the actual measurement results at hand.

Invariant and reliable quality-assured measurement results based on questionnaires require principally two actions when analyzing responses: (1) raw data from questionnaires (here: respondents rated degree of agreement to each statement) are always ordinal [7], and need to be transformed on to a common measurement interval scale on which distances have quantitative meaning [8,9], and (2) raw questionnaire response data are a mix of person leniency and item quality, which need to be estimated separately.

RMT is a means of performing both of these actions: measurement data on interval (in contrast to ordinal) scales can be reliably analyzed with all the regular statistical tools and metrics, and separate estimates of person leniency and item quality enable metrological references for comparability, in much the same way as mass standards can only be established with separately calibrated weighing machines [10].

RMT was developed by the Danish mathematician Georg Rasch in the mid-20th century with the intention to enable invariant and individual comparisons, based on the same underlying principles as physical measurements [8]. In metrological terms, RMT can be understood as modeling a measurement system in which each item of a questionnaire represents a characteristic of an object to be measured (here: the quality demand value δ), and each person responding to the questionnaire acts as a measurement instrument (with a certain respondent leniency θ) when providing a system response to the questionnaire (here: respondents’ rated degree of agreement to each statement) [9].

According to RMT, data are evaluated against a mathematical model for guiding the construction of stable linear measures from raw data [8]. In the simplest, dichotomous case, transformation of questionnaire responses is made with a logistic regression function where *P_success_* is the probability of making a *correct* binary response:

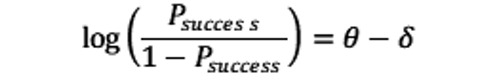



Rasch modeled the log-odds of a *yes or pass* response to a *no or fail* response as a simple linear difference between person leniency (θ) and item quality (δ). This dichotomous model can be extended to a polytomous model (ie, Likert scales with multiple ratings) [11], which has been used in this study.

### Statistical Analysis

The measurement properties of the new mHealth Satisfaction Questionnaire were evaluated according to RMT, which estimated each participant’s level of leniency (how easily satisfied they were) and each item’s level of quality (the ability of each aspect of the app to make the user satisfied) by making a logistic regression to the complete set of responses of the whole cohort to all items of the questionnaire. This was done with the software Rasch Unidimensional Measurement Model (RUMM) 2030. To ensure that this logistic regression satisfied fundamental measurement properties, the analysis focused on the requirements *response category functioning*, *targeting and reliability,* and *model fit* [12,13].

#### Response Category Functioning

To evaluate the monotonicity of item response categories, the threshold orders were evaluated. Ratings on each item should be consistent with the metric estimate of the underlying construct, that is, ordered from low to high degree of agreement. This was completed as a first step, and where needed, categories were collapsed when disordered thresholds occurred [13].

#### Targeting and Reliability

Person locations should ideally mirror the item locations. Comparing the mean person location with the mean item location (ie, 0 logits) gives an indication to whether a person is off centered from the items [13]. Moreover, to evaluate the ability to successfully furnish separate estimates of each respondent’s level of leniency, the Person Separation Index (PSI) was used to estimate reliability. Reliability, that is, the scale’s ability to discriminate correctly between person leniency, was interpreted as follows: zero (0) indicates total uncertainty and one (1) implies no uncertainty, a result >0.70 is required for group assessments and >0.85 for individual high-stake evaluations items [13,14]. A person separation reliability of 0.8 indicates that measurement uncertainty is not more than one half of the total standard deviation observed [15].

#### Model Fit

Several fit statistics were evaluated including; fit residuals, χ^2^, item characteristic curve (ICC), differential item functioning (DIF), local dependency, and unidimensionality. We used the following guidelines:

1. A residual is the difference between a person’s observed score on each item of the mHealth Satisfaction Questionnaire and the expected value derived from the RMT analysis. The mean residual is recommended to be close to zero (0) and standard deviations (SDs) close to one (1). At the same time, the individual item fit residuals should be within the range of −2.50 to +2.50 [12].
2. χ^2^ tests, which evaluate the difference between the observed and expected item responses, should ideally not be statistically significant (after Bonferroni correction) [11,12].
3. ICCs are graphical indicators of fit. They can be used to complement the interpretation of the fit residuals and χ^2^ probabilities. For ICC graphs, the dots of the class intervals should follow the ICC to support good fit [12].
4. DIF analyses are used to evaluate to which extent item responses are influenced by external factors, that is, item function should be similar across different groups and should ideally be nonsignificant (after Bonferroni correction) [16]. Both uniform and nonuniform DIF were tested for intervention group, age group, and gender. Age groups were created according to the following: *younger* corresponded to ≤39 years; *middle age* corresponded to 40 to 54 years; and *older* corresponded to ≥55 years.
5. Local dependency was evaluated according to a relative cut off of 0.2 above the average correlation [17,18]. To deal with local dependency, sets of items were grouped into new polytomous items, that is, *super items* with scores ranging from zero (0) to the maximum of the sum of the scores of the included items [19].
6. The Smith method for testing unidimensionality was applied [20]. This means that the first residual factor obtained in a principal component analysis is used to define two subsets of items by dividing them into positively and negatively correlated items. Thereafter, person estimates for each subset were compared using an independent *t* test. To support unidimensionality, the percentage of respondents outside the range −1.96 to 1.96 should not exceed 5%.

## Results

### 14 Item Version

We found disordered thresholds for all except one item in the questionnaire (*It was good to use*). This was, however, resolved by collapsing the response options. The optimal rescoring occurred when response categories 2, 3, and 4 were collapsed into one category. This was also done for the item with no disordered thresholds, as category probability curves showed close to disordered thresholds. Consequently, a 3-step scale was used for the remaining analyses, which was similar for all 14 items.

Comparisons of mean person location (0.60 logits; SD 1.13) and the mean item location (fixed to 0 logits, SD 0.71) indicated that most persons’ leniency levels were off centered with respect to the item quality locations. The PSI was 0.79, that is, the scale’s ability to discriminate correctly between person leniency was acceptable for group comparisons but not for individual evaluations. In analysis of item hierarchy, the items were ordered in a logical line from the easiest demands for product quality (eg, *It was too time consuming* or *It was easy to use*) to the more demanding qualities of the product (eg, *It has helped me to understand the benefits of improving my lifestyle habits*).

**Figure 2:**
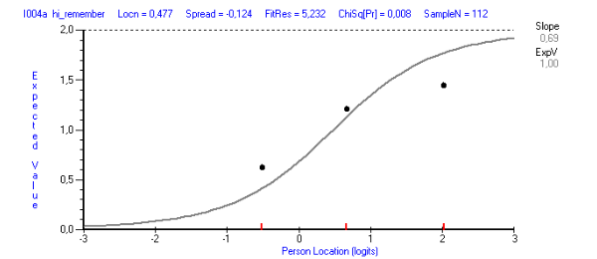
Item characteristic curve (ICC) showing a line with the expected response (predicted from the model) and the dots corresponding to the observed response. The illustration shows how the dots deviated from the ICC for the item *It has been difficult to remember to use it.*

**Figure 3:**
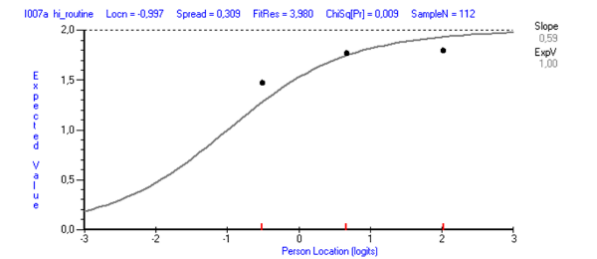
Item characteristic curve (ICC) showing a line with the expected response (predicted from the model) and the dots corresponding to the observed response. The illustration shows how the dots deviated from the ICC for the item *It interrupted me in my daily activities.*

The mean of the fit residuals was −0.45 (SD 1.4), indicating room for improvement in terms of fit to the RMT model. Nevertheless, the item fit statistics were satisfactory for all except two items, *It has been difficult to remember to use it* and *It interrupted me in my daily activities* (fit residuals 5.25 and 3.98, respectively; Table 1). None of the items showed statistically significant χ^2^, although, by studying the ICC, deviating dots were present for the two items with high fit residuals (Figures 2 and 3).

**Table 1:** Summary item statistics of the analyses for the version with 14 items.

Items	Location	2 SE	Fit residuals	Chi-square (*df*=2)	*P* value
It was a disturbance	−1.12	0.36	−0.03	1.3	.52
It interrupted me in my daily activities	−1.00	0.35	*3.98* ^a^	9.5	.01
It was too time consuming	−0.94	0.35	−0.17	0.5	.79
The introduction of how to use it was sufficient	−0.45	0.37	1.14	3.2	.21
It was boring to use	−0.36	0.30	0.61	1.4	.50
It was easy to use	−0.29	0.36	−0.83	2.2	.33
It was good to use	−0.01	0.43	−1.16	4.6	.10
The time spent using it has been acceptable	0.12	0.40	−0.70	0.8	.68
I can recommend it to others	0.40	0.36	−0.87	2.7	.26
It has been difficult to remember to use it	0.48	0.27	*5.23*	9.6	.01
It has motivated me to change my lifestyle habits	0.72	0.38	−1.91	5.8	.06
It has helped me to understand the benefits of improving my lifestyle habits	0.74	0.39	−1.99	5.3	.07
It has helped me to understand how I need to change my lifestyle habits	0.78	0.38	−2.20	4.1	.13
It has helped me set personal goals for my lifestyle habits in a way that I could not have done on my own	0.94	0.37	−1.55	3.1	.21

^a^Fit residuals in *italic* indicate misfit +2.5.

There was no significant DIF present, neither uniform nor nonuniform, for any of the person factors (intervention group, age group, or gender). In total, 16 of 91 residual correlations failed to meet the relative cut off (0.14). A clear pattern of three distinct clusters was apparent, reflecting negative experiences, positive experiences, and lifestyle consequences of using the mHealth solution, respectively (Table 2). In addition, local dependency was also shown between the two items with unsatisfactory fit residuals. A *t* test revealed that 17.9% (20/112) of the respondents were outside the desired range of −1.96 to 1.96.

**Table 2:** Summary item statistics of the analyses for the version with 12 items.

Items	Location	2 SE	Fit residuals	Chi-square (*df*=2)	*P* value	Testlet
It was a disturbance	−1.20	0.37	1.97	2.30	.32	1
It interrupted me in my daily activities	—^a^	—	—	—	—	—
It was too time consuming	−1.03	0.36	0.97	2.33	.31	1
The introduction of how to use it was sufficient	−0.53	0.38	1.46	0.87	.65	2
It was boring to use	−0.44	0.30	2.52	2.96	.23	1
It was easy to use	−0.37	0.37	−0.90	1.58	.45	2
It was good to use	−0.08	0.45	−1.38	2.86	.24	2
The time spent using it has been acceptable	0.09	0.41	−0.77	0.66	.72	2
I can recommend it to others	0.38	0.37	−0.82	1.98	.37	2
It has been difficult to remember to use it	—	—	—	—	—	—
It has motivated me to change my lifestyle habits	0.72	0.39	−2.02	3.93	.14	3
It has helped me to understand the benefits of improving my lifestyle habits	0.73	0.40	−2.44	3.01	.22	3
It has helped me to understand how I need to change my lifestyle habits	0.78	0.40	−2.66	1.98	.37	3
It has helped me set personal goals for my lifestyle habits in a way that I could not have done on my own	0.94	0.38	−1.01	1.11	.57	3

^a^The items have been removed.

### 12 Item Version

**Figure 4:**
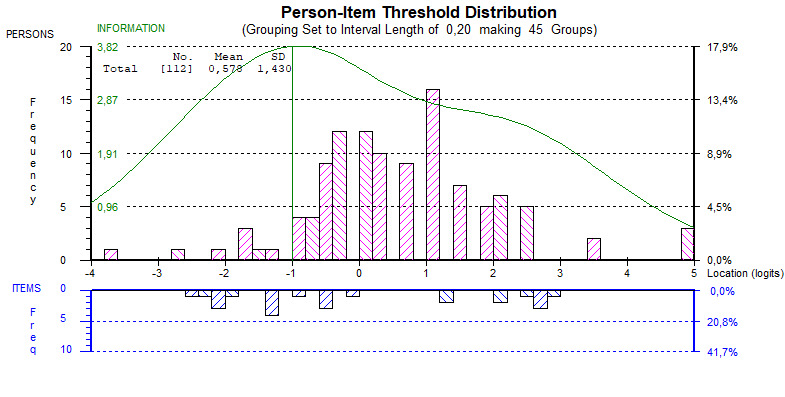
Person-item threshold histograms for the 12-item version. Upper histogram (pink bars) shows person measurements reflecting lower leniency with the Health Integrator app to the left, and higher leniency with the Health Integrator app to the right, that is, the most lenient persons are to the right. The lower histogram (blue bars) shows item threshold estimates reflecting lower quality demands to the left and higher quality demands to the right. This implies that it was easier to agree to statements at the lower end corresponding to negative experiences of using the mobile health (mHealth) app compared with the less easy items to agree with at the upper end, corresponding to lifestyle consequences of using the mHealth app.

By qualitatively studying the findings and taking the statistics into consideration, it was clear that the items *It has been difficult to remember to use it* and *It interrupted me in my daily activities* did not fit the scale. As a next step, we removed these items and reanalyzed the data. This slightly reduced the targeting (Figure 4) and PSI (from 0.79 to 0.78), but at the same time improved the fit statistics (Table 2). On the basis of the results from the analyses, an updated version, version 2, of our mHealth Satisfaction Questionnaire is presented in Multimedia Appendix 2.

As shown in Figure 4, there is a gap in items for quality between 0 and 1 logits for the threshold distribution (lower histogram, blue), which could lead to poorer measurement accuracy where most of the respondents are located (upper histogram, pink). There also seems to be room toward the upper end of the scale to include items making more demands for quality using the mHealth app items, and similarly, some less demanding items at the lower end of the quality scale.

Two items showed fit residuals just outside the range −2.5 to +2.5 (*It has helped me to understand how I need to change my lifestyle habits* −2.66; and *It was boring to use* 2.51; Table 2). However, these items showed neither statistically significant χ^2^ nor deviating dots from the ICC. Again, a high number of local dependencies (13 of 66 correlations above the relative cut off 0.13) was found, with similar patterns as reported above, and 16.1% (18/112) of the respondents were still outside the desired range of −1.96 to 1.96 when examining unidimensionality. To deal with local dependency, the clustered items were grouped into three testlets (Table 2), and the analyses were repeated. This, however, resulted in disordered thresholds for all testlets. This could not be solved without affecting the content and the satisfactory fit statistics reported above.

## Discussion

### Principal Findings

We present the initial development of a questionnaire, the mHealth Satisfaction Questionnaire, for assessing user satisfaction with mHealth apps and demonstrate a metrological way of evaluating and redesigning the questionnaire’s ability to assess this. By applying Rasch analysis, we can better understand the limitations of the questionnaire, as well get guidance about how to revise and improve the questionnaire.

Disordered thresholds indicate that respondents had difficulty in discriminating between the given response options [21]. Unsatisfactory statistics for the monotonicity of item response categories might, however, be a consequence of the small sample size [22]. We solved this by collapsing the 5-point Likert scale into a 3-point Likert scale. With too many response alternatives, there is always a possibility of collapsing response options, but the other way around, that is, splitting responses into two or more categories, cannot be done.

By studying the clusters from the residual correlations used for testlets, three underlying dimensions emerged representing negative experiences of using the mHealth app (testlet 1), positive experiences of using the mHealth app (testlet 2), and lifestyle consequences of using the mHealth app (testlet 3). Given the lack of unidimensionality, it may be questionable to create a single score for a higher ordered assessment of satisfaction with mHealth apps. On the other hand, having a too hardline data–driven approach is not without risk [23]. By considering the clusters that emerged, it was clarified that the items were ordered in a logical hierarchy from the easiest demands for product quality to the more demanding qualities of the product. For mHealth apps, this means that the hierarchy is going from not having negative effects, through positive experiences in the everyday usage, to having a positive impact on lifestyle. Consequently, with those distinct steps in the hierarchy, it can be of importance to specify thresholds or requirement for different types of mHealth apps or to provide guidance for mHealth app developers on what actions are necessary to improve user satisfaction or to ensure higher user satisfaction.

Our analysis showed slightly off-centered persons to the items and that there were some gaps in both item locations and threshold locations. Together with a reliability of 0.78, this implies that the persons’ leniency values are measured with a low precision. This indicates a need for the inclusion of additional items to close the gaps and create a more granulated measure with less measurement uncertainties [12,24]. Adding more items might also help to remove local dependency, as assessments of local dependency seem to be less reliable when there are fewer than 20 items [18]. Future studies exploring which additional items could be brought in, perhaps through respondent interviews to ensure the content validity, may be of value in making sure that the questionnaire captures the full range of concepts of interest.

### Limitations

There are some methodological limitations to bear in mind when interpreting the results. Despite a response rate from our randomized controlled trial of over 80%, the sample size (n=112) could still be considered small. To reach a reliability of 0.8, which is considered an acceptable metrological convention for measurement uncertainties [15], another 14 respondents would have been needed, according to the Spearman-Brown prediction formula [25]. However, in the early stages of methodological work, small sample sizes could be considered acceptable as *convenience samples* for explorative purposes [22].

Another limitation is the frame of reference, that is, that the measurement properties of the mHealth Satisfaction Questionnaire are only evaluated when applied to one unique mHealth app and in one context. However, this is a first step in developing and assessing the measurement properties of the mHealth Satisfaction Questionnaire. With our promising results and suggested improvements for future work, we would recommend that the measurement properties of a future refined version of the mHealth Satisfaction Questionnaire are evaluated further. In particular, DIF, which did not vary with age, gender, or intervention in our sample, would be of interest to study in groups that have used different mHealth apps.

### Comparison With Prior Work

Already in 1986, long before eHealth and later on mHealth had made their entries, Nicell et al [26] designed a questionnaire to measure attitudes toward computers. It included 20 statements like *I feel intimidated by computers* and *Computers are bringing us to a bright new era* with response alternatives on a 5-point Likert scale. Usability, including efficiency, efficacy, and satisfaction, is today a widely accepted metric in many fields and is the focus of several standards and regulations. Initially applied to visual display terminals [27], these standards are now slowly being introduced into medical device and user interface regulations [28,29] and are regulated and promoted by the US Food and Drug Administration [30].

Despite the fact that computers, eHealth, and mHealth have become such an integrated part of everyday life and that mHealth apps commonly act as medical devices, surprisingly little research has been conducted into finding valid and reliable ways to assess user satisfaction with these digital solutions. Besides, different rating scales are increasingly used as outcome measures in clinical studies. Our proposed mHealth Satisfaction Questionnaire, or similar scales evaluated in rigorous ways, can facilitate and guide future development of mHealth solutions and be included in comprehensive usability tests. In addition, such questionnaires may even be of great importance for the accreditation process of mHealth apps and medical technology products.

Our mHealth Satisfaction Questionnaire is an adapted version of similar questionnaires found in the mHealth literature [3,4]. However, how the first versions were developed measurement, and psychometric properties of the previous questionnaires evaluated are not described. They are also more context specific than generic. On the other hand, the mHealth Satisfaction Questionnaire presented in this study provides both an evaluation of its measurement properties and a questionnaire for generic usage in mHealth solutions.

The RMT is considered conceptually and theoretically preferable compared with classical test theory (CTT), both in designing and evaluating rating scales. Limitations with CTT include that (1) data generated are ordinal, (2) scores are scale dependent, (3) scale properties are sample dependent, and (4) data are only suitable for group studies [31]. On the other hand, RMT provides separate estimates of person and item attribute values and their scaling on a common interval logit scale. Moreover, there is a growing interest in RMT in the health care literature [32]. For instance, it has been used to compare measurement performance of questionnaires in diverse areas such as depression in a sample with diverse severity of emotional distress [24], physical and psychological impact of multiple sclerosis [9], and quality of life in sarcoidosis [33], but to our knowledge, psychometric evaluations of rating scales for satisfaction assessment with mHealth have never been conducted. Previously, the Rasch approach to evaluating full usability has only been applied on a few occasions, including in the analysis of Web usability [34] and incontinence product usability [35].

### Conclusions

Taken together, although there is room for improvement, our mHealth Satisfaction Questionnaire gives a new possibility to measure user satisfaction with mHealth. In times where mHealth and digital solutions are given more attention, the mHealth Satisfaction Questionnaire could be an important piece to ensure usability assessments and improve development of mHealth solutions. This paper provides the initial work and suggests further development, where additional items are examined, larger samples are used, and the mHealth Satisfaction Questionnaire is tested for other eHealth apps and in other contexts of use.

## Appendix

Multimedia Appendix 1 The mHealth Satisfaction Questionnaire, version 1.

Multimedia Appendix 2 The mHealth Satisfaction Questionnaire, version 2.

## Abbreviations

CTT: classical test theory
DIF: differential item functioning
eHealth: electronic health
ICC: item characteristic curve
mHealth: mobile health
PSI: Person Separation Index
RMT: Rasch measurement theory
