# Supplementary material for: A Questionnaire for Assessing User Satisfaction With Mobile Health Apps: Development Using Rasch Measurement Theory
Source: JMIR Mhealth Uhealth. 2020 May 26;8(5):e15909. doi: 10.2196/15909 (PMC7284402; doi:10.2196/15909)
Supplement: Multimedia Appendix 2 [file mhealth_v8i5e15909_app2.docx]

**Appendix 2. The mHealth Satisfaction Questionnaire, version 2**

|  | ***Strongly Disagree***  *1* | *2-3-4* | ***Strongly Agree***  *5* |
| --- | --- | --- | --- |
| **What did you think about using the health app?** | | | |
| It was easy to use |  |  |  |
| It was good to use |  |  |  |
| The time spent using it has been acceptable |  |  |  |
| The introduction of how to use it was sufficient |  |  |  |
| It was too time consuming |  |  |  |
| It was boring to use |  |  |  |
| It was a disturbance |  |  |  |
| I can recommend it to others |  |  |  |
| **How did you experience the health app?** | | | |
| It has motivated me to change my lifestyle habits |  |  |  |
| It has helped me to understand the benefits of improving my lifestyle habits |  |  |  |
| It has helped me to understand how I need to change my lifestyle habits |  |  |  |
| It has helped me set personal goals for my lifestyle habits in a way that I could not have done on my own |  |  |  |
